# Subcellular Compartmentalization of Survivin is Associated with Biological Aggressiveness and Prognosis in Prostate Cancer

**DOI:** 10.1038/s41598-020-60064-9

**Published:** 2020-02-24

**Authors:** Jan K. Hennigs, Sarah Minner, Pierre Tennstedt, Rolf Löser, Hartwig Huland, Hans Klose, Markus Graefen, Thorsten Schlomm, Guido Sauter, Carsten Bokemeyer, Friedemann Honecker

**Affiliations:** 1 Department of Internal Medicine II – Oncology, Hematology, Bone Marrow Transplantation and Pneumology, University Medical Center Hamburg-Eppendorf, Hamburg, Germany; 2 Institute of Pathology, University Medical Center Hamburg-Eppendorf, University Medical Center Hamburg-Eppendorf, Hamburg, Germany; 3 Martini Clinic, Prostate Cancer Center, University Medical Center Hamburg-Eppendorf, Hamburg, Germany; 4 Department of Urology, Charité-Universitätsmedizin, Berlin, Germany; 5 Tumor and Breast Center ZeTuP, St. Gallen, Switzerland

**Keywords:** Immunochemistry, Microarrays, Predictive medicine, Prostate cancer, Surgical oncology

## Abstract

The role of subcellular survivin compartmentalization in the biology and prognosis of prostate cancer is unclear. We therefore investigated subcellular localization of survivin in more than 3000 prostate cancer patients by quantitative immunohistochemistry and performed transcriptomics of 250 prostate cancer patients and healthy donors using publicly available datasets. Survivin (BIRC5) gene expression was increased in primary prostate cancers and metastases, but did not differ in recurrent vs non-recurrent prostate cancers. Survivin immunohistochemistry (IHC) staining was limited exclusively to the nucleus in 900 prostate cancers (40.0%), and accompanied by various levels of cytoplasmic positivity in 1338 tumors (59.4%). 0.5% of prostate cancers did not express survivin. Nuclear and cytoplasmic survivin staining intensities were strongly associated with each other, pT category, and higher Gleason scores. Cytoplasmic but not nuclear survivin staining correlated with high tumor cell proliferation in prostate cancers. Strong cytoplasmic survivin staining, but not nuclear staining predicted an unfavorable outcome in univariate analyses. Multivariate Cox regression analysis showed that survivin is not an independent prognostic marker. In conclusion, we provide evidence that survivin expression is increased in prostate cancers, especially in metastatic disease, resulting in higher aggressiveness and tumor progression. In addition, subcellular compartmentalization is an important aspect of survivin cancer biology, as only cytoplasmic, but not nuclear survivin accumulation is linked to biological aggressiveness and prognosis of prostate cancers.

## Introduction

Survivin, a 16.5  kDa protein, is the smallest member of the *Inhibitor of Apoptosis* (IAP) protein family^1^. It exists in three distinct subcellular pools, namely the cytoplasm, mitochondria, and the nucleus^1,2^. Established molecular features of survivin comprise inhibition of apoptosis, promotion of cell proliferation as a central regulator of spindle formation, and promotion of tumor angiogenesis^3,4^. Survivin expression and function is regulated by transcriptional, post-transcriptional, and post-translational mechanisms like ubiquitination and phosphorylation^1^. Yet another level of complexity of survivin signaling is achieved by expression of different splice variants that can exert opposing apoptotic as well as anti-apoptotic functions^5,6^.

Survivin expression has been reported in a wide variety of normal and fetal tissues^7^. Increased survivin expression has been found in various malignancies including cancers of the lung, prostate, pancreas, colon, breast, and high-grade Non-Hodgkin lymphomas. Especially in lung, colorectal, oral squamous cell, and breast cancer, an association between survivin expression and biologically aggressive cancer subtypes and thus poor prognosis has been established^4^. Survivin overexpression has been shown to strongly inhibit cell death in a multitude of cells^8^. Additionally, experimental downregulation of survivin led to increased spontaneous cell death, an enhanced response to apoptotic stimuli such as chemotherapy, and reduced tumor angiogenesis^4,8^.

In normal tissues, survivin expression is transcriptionally repressed by the tumor suppressor p53^9,10^. Mutated p53 loses the ability to repress survivin transcription^11^. In prostate cancer, p53 is mutated in a subset of biologically aggressive tumors, which is associated with a significantly increased risk of progression after radical prostatectomy^12^.

Total survivin expression in prostate cancers has previously been investigated by PCR and IHC, and protein expression has been described in approx. 70–80% of cases, mainly in more aggressive/more advanced tumors^13–16^. A positive correlation of survivin protein expression (assessed by Western Blotting) and higher Gleason Scores was described in samples of 73 prostate cancer patients^17^. In a case-control study of over 1000 Chinese men, Chen *et al*. described a positive correlation between the prevalence of a certain polymorphism in the survivin promoter and the risk to develop prostate cancer^18^. Even though the biological background is not clearly defined yet, this finding suggests a role of survivin early in the pathogenesis and progression of prostate cancer. Interestingly, the subcellular localization of survivin appears to be relevant in prostate cancer. Nuclear survivin staining has been linked to good prognosis in a small study analyzing 68 patients treated within a phase III trial, whereas cytoplasmic overexpression was associated with local progression of the tumor^19^.

All these findings have stimulated interest in survivin as a potential prognostic marker and therapeutic target in prostate cancer^14–16,19,20^. To clarify the clinical significance of survivin mRNA and protein expression as well as subcellular compartmentalization in prostate cancer, we analyzed survivin expression by IHC and transcriptomics in samples of more than 3000 patients with clinically and biologically well-characterized prostate cancers, and data of 250 human prostate cancer patients and healthy donors, respectively.

## Results

### Survivin mRNA expression in cancerous and non-cancerous prostate tissue

Using the GEO GDS2545 dataset, we compared mRNA expression of the survivin gene BIRC5 in normal prostate tissue from healthy donors, normal prostate tissues adjacent to primary prostate cancer, primary cancers, and prostate cancer metastasis in an unpaired fashion.

**Figure 1:**
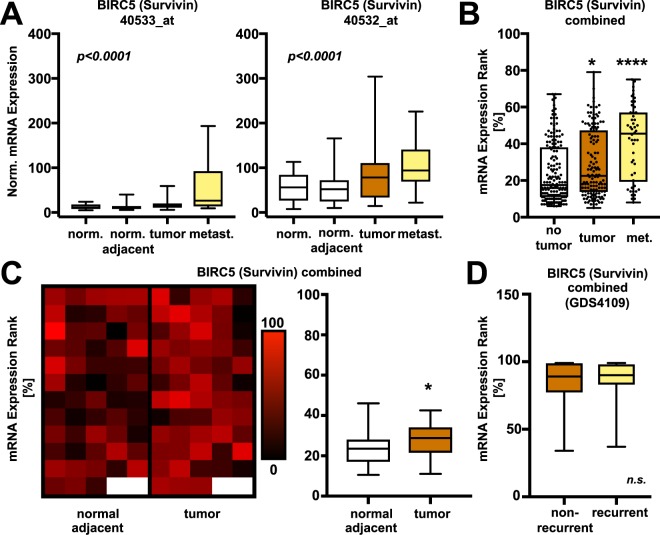
Survivin mRNA expression in healthy prostate tissues and prostate cancers. Gene expression analysis of two independent probes for BIRC5 (Survivin) shows higher BIRC5 mRNA expression in primary prostate cancers (tumor) and prostate cancer metastases (met.) compared with normal adjacent prostate tissues (norm. adjacent) and tissues from healthy donors (norm, n = 171, both ANOVA p < 0.0001; 50533_at:: normal vs. tumor: p = 0.4447, normal vs. metastasis: p < 0.0001, tumor vs. metastasis: p < 0.0001; 40532_at: normal vs. tumor: p = 0.0499, normal vs. metastasis: p = 0.0013, tumor vs. metastasis: p = 0.0277, Holm-Sidak posthoc analysis, (**A**)). Combined expression ranks analysis reveals higher BIRC5 levels in prostate cancer samples (tumor, p < 0.05) and prostate cancer metastases (met., p < 0.0001) compared to cancer-free prostate samples (no tumor, **B**, all ANOVA with Holm-Sidak posthoc test). A heatmap of relative BIRC5 mRNA expression in prostate cancer tissue (tumor) and the surrounding adjacent normal prostate per individual patient (box) is shown (**C**, left panel). Per individual patient, prostate cancers show higher BIRC5 mRNA expression than the corresponding adjacent tumor-free tissue (**C**, right panel, p < 0.05, Wilcoxon matched-pairs signed rank test). BIRC5 expression did not differ in recurrent vs. non-recurrent prostate cancers (**D**, p  > 0.05, n  =  39 vs. 40 patients, Mann-Whitney test). Standardized expression values and expression ranks were extracted from the identified GEO datasets GDS2545 and GDS4109 and compared as described in the Methods section with the statistical tests given in parenthesis (data are median ± range; *  p < 0.05 vs. corresponding control, **** p < 0.0001 vs. corresponding control).

Two separate BIRC5 probes (40532_at and 40533_at) were available for analysis, and both showed significantly differential expression across tissues (Fig. 1A, 50533_at:: normal vs. tumor: p = 0.4447, normal vs. metastasis: p < 0.0001, tumor vs. metastasis: p < 0.0001; 40532_at: normal vs. tumor: p = 0.0499, normal vs. metastasis: p = 0.0013, tumor vs. metastasis: p = 0.0277, Holm-Sidak posthoc analysis, (**A**)). Pooled analysis revealed a significant increase of BIRC5 mRNA in prostate cancers (p < 0.05) and prostate cancer metastases (p < 0.0001) compared to tissues from healthy donors or from adjacent normal prostate tissues combined (=no tumor, Fig. 1B). This was also the case when comparing BIRC5 expression in prostate cancers with the corresponding normal adjacent tissues from the same patient using paired analysis (p  =  0.0126, n  =  58 patients, Fig. 1C, right panel). In total, prostate cancers from 41 out of 58 matched patients (70.7%) showed increased BIRC5 expression compared to corresponding normal adjacent tissues (Fig. 1C, left panel). There was no difference in BIRC5 mRNA expression between recurrent and non-recurrent prostate cancers (GDS4109 dataset, p  =  0.71, Fig. 1D).

### Survivin protein expression in normal prostate tissue and prostate cancer

**Figure 2:**
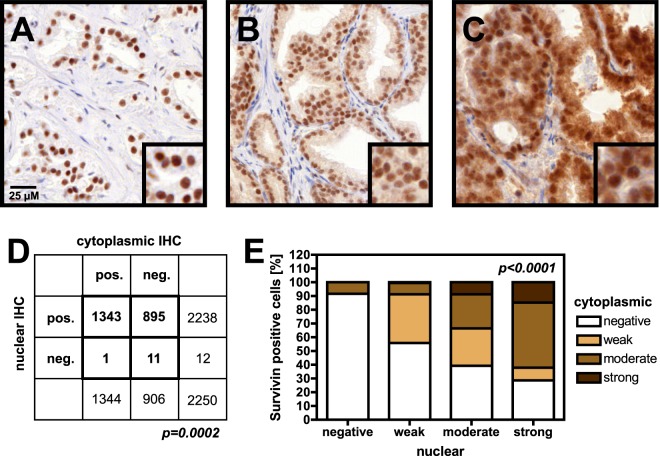
Survivin staining pattern and intensities of selected tissue microarray cores. Microphotographs (and magnifications thereof) showing nuclear only (**A**) as well as both nuclear and additional cytoplasmic (**B**,**C**) survivin staining of different intensities in prostate cancer cells. Comparisons of survivin-positive (pos.) or negative (neg.) prostate-cancer samples by subcellular localization are shown in (**D**, p = 0.0002). Pearson’s *χ*^2^ test). Staining intensities and frequencies by subcellular localization (cytoplasmic IHC relative to nuclear IHC) are shown in (**E**, p < 0.0001, Pearson’s *χ*^2^ test).

For quantitative IHC, a total of twelve cores from three different TMA blocks containing normal prostate tissue without presence of cancer cells were available for analysis. In normal prostate epithelium, strong nuclear immunostaining for survivin was present in every single sample (=100%, Supplementary Fig. 1). No nuclear staining was detected in stroma cells. No cytoplasmic staining was detected in any of the normal tissue samples. The group of interpretable cancer cases encompassed 2,250 TMA cores with prostate cancer cells (69.0% of all cores). Survivin staining (nuclear and/or cytoplasmic) was seen in 2,238 (99.5%) of interpretable prostate cancer samples (Fig. 2A–C). Nuclear survivin staining was detected in all but twelve cases (0.5%). Staining was limited exclusively to the nucleus in 895 cases (39.8%). Nuclear survivin staining was accompanied by various levels of cytoplasmic positivity in 1343 tumors (59.7%). In a single case staining was exclusively cytoplasmic (Fig. 2D). No significant heterogeneity of expression was observed within tumor samples. In general, cytoplasmic and nuclear staining was rather weak in all samples. For statistical purposes, four groups were defined according to intensity and localization of survivin staining: nuclear staining alone (n = 906; 40.3%), weak cytoplasmic staining (n = 532; 23.6%), moderate cytoplasmic staining (n = 619; 27.5%), and strong cytoplasmic staining (n = 193; 8.6%). Nuclear and cytoplasmic staining intensities showed a direct correlation (p < 0.0001, Fig. 2E and Supplemental Table 1).

### Correlation of survivin expression with clinico-pathological parameters

Tumor phenotype and clinical features were correlated with results of survivin staining. A highly significant correlation of cytoplasmic survivin staining was observed with higher Gleason Scores and advanced pT stages (p < 0.0001 each, Table 1). The same correlations were found for nuclear staining intensities (Supplemental Table 2).

**Table 1:** Survivin staining intensities and frequencies in patients undergoing radical prostatectomy.

Parameter	Variable	n =	nuclear only (%)	cytoplasmic			P value
				weak (%)	moderate (%)	strong (%)	
Gleason score	≤3 + 3	891	46.2	23.3	23.3	7.1	<0.0001
	3 + 4	950	35.6	26.2	30.1	8.1	
	4 + 3	243	36.2	18.1	30.9	14.8	
	≥4 + 4	48	29.2	16.7	37.5	16.7	
pT category	pT2	1,329	44.6	22.5	26.3	6.6	<0.0001
	pT3a	474	31.0	26.6	30.4	12.0	
	pT3b	296	33.5	24.7	28.7	13.2	
	pT4	32	40.6	34.4	21.9	3.1	
Surgical Margin Status	Negative	1,663	40.2	23.8	26.9	9.1	0.2497
	Positive	466	39.1	24.3	30.0	6.7	
Pre-operative PSA [ng/ml]	<4	320	40.9	25.0	27.5	6.6	0.4709
	4–10	1,119	41.6	23.2	27.1	8.0	
	10–20	473	37.2	23.5	28.5	10.8	
	>20	184	37.0	27.2	28.8	7.1	

Deviations from total are due to missing data in the subcategories.). Pearson’s χ^2^ test.

### Correlation of survivin staining with Ki67 IHC

**Figure 3:**
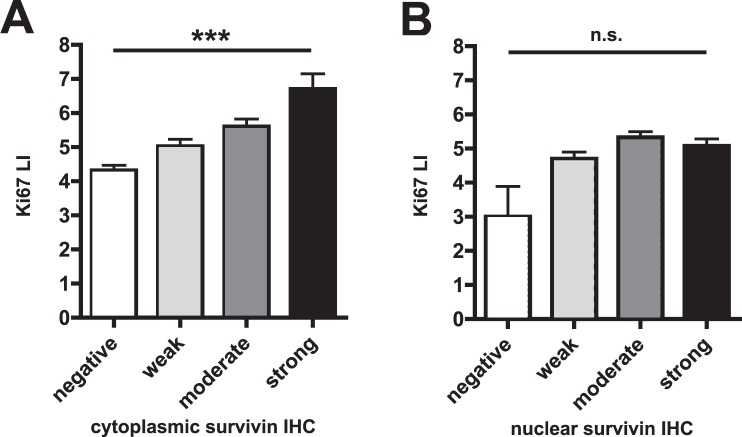
Association of Ki67 Labeling Index (LI) with subcellular survivin staining. Cytoplasmic but not nuclear survivin immunohistochemistry intensities correlate with the percentage of Ki67 positive tumor cells in prostate cancer tissue (Ki67 LI, (**A**) p < 0.0001, (**B**) p > 0.05, all ANOVA with Holm-Sidak posthoc test).

On the protein level, cytoplasmic but not nuclear staining was strongly associated with cancer cell proliferation, determined by Ki67 labeling index (Fig. 3A,B; p < 0.0001 and p = 0.06, respectively). Ki67 labeling data was available from a previous analysis on the same TMA^21^.

### Survivin compartmentalization and biochemical cancer recurrence, development of metastatic disease, and cancer-specific survival

Using Kaplan-Meier analyses, an association of established clinical and pathological factors with PSA recurrence, time to onset of metastatic disease after radical prostatectomy, and cancer-related survival was confirmed (Table 2).

**Table 2:** Associations of pathological parameters of prostate cancers samples with time to biochemical (PSA) recurrence, onset of metastasis, and cancer-specific survival in patients after radical prostatectomy.

Parameters	Log-Rank test (Kaplan-Meier analysis)					
	PSA recurrence		Cancer-specific survival		Onset of metastastic disease	
	χ^2^	P value	χ^2^	P value	χ^2^	P value
Pre-operative PSA [ng/ml]	185.0	<0.0001	11.4	0.0097	21.9	<0.0001
Gleason score	597.9	<0.0001	84.6	<0.0001	153.3	<0.0001
pT category	611.7	<0.0001	63.7	<0.0001	102.3	<0.0001
pN category	335.6	<0.0001	10.6	0.0143	34.5	<0.0001
Surgical margin status	159.5	<0.0001	20.2	<0.0001	25.4	<0.0001

**Figure 4:**
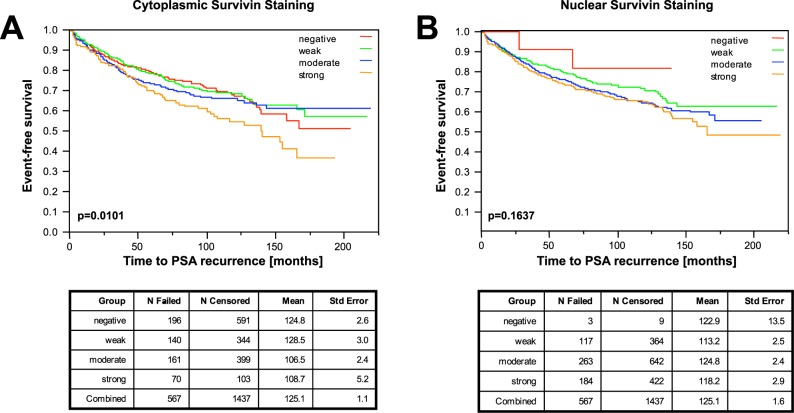
Association of subcellular survivin staining with PSA-free survival after prostatectomy. Kaplan-Meier curves of immunohistochemical staining intensities show the influence of the subcellular survivin distribution on PSA recurrence: strong cytoplasmic staining is associated with an impaired PSA-free survival (**A**, p = 0.0101, Log-Rank test), whereas nuclear survivin staining is not (**B**, p = 0.1637, Log-Rank test).

Cytoplasmic survivin staining intensity was significantly associated with biochemical relapse (earlier PSA recurrence) (p = 0.0101, Fig. 4A), but not with onset of metastatic disease (p = 0.18), and cancer-related survival (p = 0.08).

Nuclear survivin staining intensities did not significantly correlate with biochemical recurrence (p = 0.16, Fig. 4B), onset of metastatic disease (p = 0.18), or cancer-related survival (p = 0.05).

### Multivariate analysis

Multivariate Cox regression analyses were performed including Gleason Score, surgical margin status, pT stage, pre-operative PSA levels, nuclear p53 IHC, and cytoplasmic survivin staining intensity, and confirmed all established clinico-pathological parameters as independent predictive risk factors for PSA recurrence (all p < 0.0001, Table 3). However, cytoplasmic survivin staining did not emerge as independent risk factors from this analysis.

**Table 3:** Multivariate Cox regression analysis for biochemical (PSA) recurrence in patients after radical prostatectomy.

Parameter	Variable	Risk ratio	95% CI	P value
Stage	pT2	0.43	0.35–0.52	<0.0001
	pT3a	0.76	0.65–0.90	
	pT3b	1.34	1.14–1.58	
	pT4	2.21	1.64–2.88	
Gleason score	≤3 + 3	0.31	0.24–0.38	<0.0001
	3 + 4	0.70	0.61–0.82	
	4 + 3	1.77	1.50–2.10	
	≥4 + 4	2.72	2.05–3.53	
Pre-operative PSA [ng/ml]	<4	0.74	0.58–0.93	<0.0001
	4–10	0.86	0.75–1.0	
	10–20	1.15	0.99–1.34	
	> 20	1.44	1.20–1.71	
Surgical Margin Status	Negative vs. positive	0.74	0.67–0.81	<0.0001
Nuclear p53 IHC	Positive vs. negative	1.27	1.04–1.52	0.0189
Cytoplasmic Survivin IHC	Negative	1.11	0.96–1.28	0.1585
	Weak	0.89	0.75–1.04	
	Moderate	0.92	0.79–1.06	
	Strong	1.11	0.89–1.35	

## Discussion

The aim of the present study was to comprehensively assess cytoplasmic and nuclear survivin IHC in prostate cancer in correlation with well-defined clinical and pathological parameters, and to assess the potential of survivin as a prognostic factor in prostate cancer.

In healthy prostate tissue, survivin staining was absent in the cytoplasm, but was observed in the nucleus of epithelial cells. In prostate cancers, 99.4% of the samples showed positive surviving staining, with 40% of the samples showing an exclusively nuclear staining pattern. Cytoplasmic survivin staining was associated with biologically aggressive disease, i.e. higher Gleason scores, higher pathological tumor stages, and strong proliferative activity. Although strong cytoplasmic (but not nuclear) survivin staining showed an association with unfavorable clinical outcomes as determined by Kaplan-Meier-Analysis, survivin did not emerge as an independent prognostic risk factor from our multivariate Cox regression analysis.

Increase in survivin protein levels appears to be regulated on the transcriptional level, as mRNA expression of the survivin gene BIRC5 is also increased in prostate cancers.

Descriptive and functional data on the presence and role of survivin in prostate cancer published within the last years revealed highly contradictory results^13–17,19,22–24^.

Several studies, both from preclinical models and prostate cancer trials, have suggested a pivotal role of survivin in prostate cancer pathophysiology^19,25^, for instance via the survivin/TGFβ/mTORC axis in IGF-1 mediated growth^26^ or the SHARPIN/NFkB axis in survivin/livin mediated tumorigenesis and invasiveness^26,27^. This is in line with recent data showing that repression of survivin expression in prostate cancer cells exerts anti-proliferative effects *in vitro*^28^ and *in vivo*^29^.

Whereas two studies have shown an association of survivin staining with an increased risk of local progression^14,19^, another study by Kaur *et al*. could not detect any association between survivin and prognostic or clinco-pathological parameters including pT stage, grading, or cancer relapse after radical prostatectomy^16^.

Compared to our present analysis, most previous studies - in much smaller patient cohorts - have found similar survivin staining rates between 71% and 83%^13–16^. In contrast to our current work these studies have, however, mostly not distinguished between nuclear and cytoplasmic immunostaining.

Zhang *et al*. could show that nuclear overexpression was independently correlated with improved prostate cancer survival in a small prospective cohort of 68 patients with locally advanced prostate cancer within the RTOG 8610 trial^19^. In addition, the same group has presented preclinical and clinical data from 62 patients, suggesting that survivin promotes the metastatic process in prostate cancer^25^.

Depending on its subcellular localization, survivin seems to exhibit different functions. Nuclear survivin, most likely in its homodimeric form^30^, lacks anti-apoptotic potential^31^. Cytoplasmic survivin, on the other hand, usually originates from mitochondria, as it is rapidly released from mitochondria upon pro-apoptotic stimuli^32^. Once cytoplasmic, survivin interacts with another IAP protein, XIAP^33^, which is concertedly upregulated in prostate cancers^15^. The resulting survivin-XIAP heterodimers facilitate anti-proteasomal stability and inhibition of caspase-mediated apoptosis, thereby promoting tumor growth and survival^4,32,33^. In contrast, survivin downregulation has been reported to be both effective in directly inducing apoptosis and sensitizing cancer cells from various histologies (including prostate cancer) to different cytotoxic agents^34–37^.

It has been suggested that in normal tissues, survivin levels are kept low, whereas malignant transformation can lead to increased gene expression of survivin^9^. This is in line with our finding of increased survivin expression in biologically aggressive prostate cancers, both on mRNA and protein levels. Indeed, all but a single study mentioned above^16^, support these findings^11,13–15,17,19,20,22,24,25,37,38^.

In various cancers other than prostate cancer the single most important regulator of survivin expression is the prototypical tumor suppressor p53^39^. Nuclear accumulation of p53 is a risk factor for prostate cancer progression and prognosis^40^. In addition, in a study using the present dataset (among others), strong nuclear p53 immunoreactivity by IHC was strongly associated with mutations in the p53 gene, TP53. As has been shown before by multiple groups, survivin/BIRC5 is one of the few genes whose expression is transcriptionally repressed by direct binding of wildtype p53 to the survivin/BIRC5 promoter, and activation of wildtype p53 leads to cell cycle arrest or apoptotic cell death in cancer cells^9,10,41^, whereas knockdown of p53 leads to increased survivin mRNA expression (Supplemental Fig. 2). In addition, another p53-related mechanism for transcriptional repression of BIRC5 via Retinoblastoma(Rb)/E2F-family interactions has been identified^42^.

Using the androgen-refractory PC3 prostate cancer cell line Shao and colleagues could show an inverse correlation between p53 and survivin (r^2^ = 0.55) expression *in vitro* via direct inhibitory protein-protein interaction^39^. Therefore, current data suggests a central pathophysiological role for p53 in regulation of survivin function and/or expression in prostate cancer. However, the detailed mechanism of survivin dysregulation in prostate cancer has to be determined in further studies.

In summary, our study documents that survivin is present in the nuclei of normal prostate epithelial cells and prostate cancer cells. Additional expression in the cytoplasm as determined by IHC occurred in approximately 60% of prostate cancers, and showed an association with early PSA relapse in univariate analysis. Furthermore, cytoplasmic survivin was associated with features of biological aggressiveness including increased cancer cell proliferation, Gleason score and pT stage. However, in contrast to previously published studies with a less comprehensive approach and mostly smaller sample size, survivin alone did not emerge as an independent prognostic biomarker in prostate cancers, regardless of its compartmentalization.

## Methods

### Tissue microarray construction

Prostatectomy specimens were processed according to standard procedures. Sampling and constructions of the tissue microarray (TMA) have been previously described in detail^12^. The prostate cancer prognosis TMA consists of cancer samples from 3,261 patients distributed over 7 paraffin blocks. In brief, specimens from radical prostatectomies performed between 1992 and 2005 at the Department of Urology, University Medical Center Hamburg-Eppendorf were paraffin-embedded and afterwards matched with clinico-pathological data.

In all patients undergoing radical prostatectomy, prostate specific antigen (PSA) concentrations were measured quarterly in the first year followed by biannual measurements in the second and annual measurements after the third year following surgery. Biochemical recurrence was defined as a postoperative PSA of 0.2  ng/ml. Time of recurrence was defined by the first PSA value above or equal to 0.2  ng/ml. Patients without evidence of tumor recurrence were censored at last follow-up. No patient of the cohort received neo-adjuvant or adjuvant endocrine therapy.

For TMA construction, representative tissue cylinders with a diameter of 0.6  mm were punched from tumor areas of a paraffin-embedded donor tissue block and transferred to the corresponding coordinates on the recipient paraffin block in a half-automated process using precision instruments. Four-micrometer thick sections of each microarray block were transferred to adhesive slides for IHC analyses.

### Immunohistochemistry (IHC)

Freshly cut TMA sections were stained in one experiment on a single day. TMA sections were de-paraffinized followed by heat-induced antigen retrieval in an autoclave in acetate buffer pH 9.0 for 5  min. Primary polyclonal rabbit anti-Survivin antibody (RB-9245, Thermo Scientific, Fremont, CA, USA) was used in a final solution of 1:900. Survivin expression was visualized utilizing the Envision System (DAKO, Glostrup, Denmark).

Nuclear and cytoplasmic staining was evaluated separately for each spot and quantified as described previously^43,44^. In brief, staining intensity (negative = 0, weak = 1+, moderate = 2+, strong = 3+) and fraction of positive tumor cells were recorded for each tissue spot. A final score was built from these two parameters according to the following scores: Negative scores had staining intensity of 0, weak scores had staining intensity of 1+ in ≤70% of tumor cells or staining intensity of 2+ in ≤30% of tumor cells; moderate scores had staining intensity of 1+ in >70% of tumor cells, staining intensity of 2+ in >30% and ≤70% of tumor cells or staining intensity of 3+ in ≤30% of tumor cells and strong scores had staining intensity of 2+ in >70% of tumor cells or staining intensity of 3+ in >30% of tumor cells.

Ki67^21^ IHC data generated on the same TMA were available from previous studies and IHC was performed as previously published.

All TMA spots were evaluated for the presence of prostate cancer cells. Only cancer-positive cores were included in statistical analyses.

### Transcriptomic analysis

A Gene Expression Omnibus (GEO) search was conducted for human gene array datasets as previously described^45^. Two datasets were identified. GDS2545 contains RNA expression data from 65 primary prostate cancers, 63 normal tissues adjacent to prostate cancer, 25 prostate cancer metastases and 18 normal healthy prostate tissues hybridized to the Affymetrix Human Genome U95 Version 2 Array platform (GPL8300). GDS4109 contains RNA expression data from 39 recurrent and 49 non-recurrent primary prostate cancers hybridized to the Affymetrix Human Genome U133A Array platform (GPL96). Normalized gene expression values and ranks were extracted and pre-analyzed using the GEO Dataset Browser data analysis online tools and quantified locally using R (version 3.3.3), R Studio Desktop and Biobase, GEOquery and limma libraries.

### Statistical analysis

Statistical analyses were accomplished using JMP 5.0.1 software (SAS Institute Inc., Cary, NC, USA) and PRISM 7 (Graphpad Inc, La Jolla, CA, USA). Pearson’s chi-square test was used for contingency tables. Analysis of Variances (ANOVA, with Holm-Sidak posthoc analysis) was used to test the association of Ki67 labeling index, p53 accumulation, and survivin expression, all determined by IHC, and to compare mRNA expression in normal and tumor tissues when more than two groups were compared. Wilcoxon matched-pairs signed rank test was used to test mRNA expression differences between matched prostate cancer samples and adjacent normal prostate tissues from the same patient. Mann-Whitney test was used to compare expression ranks between non-recurrent and recurrent prostate cancers. Survival curves were calculated by Kaplan–Meier analysis and compared by log rank test. Multivariate analysis with Cox regression was used to test independence of clinical parameters, survivin expression and p53 accumulation as risk factors for PSA-recurrence, metastatic disease and cancer-related survival after radical prostatectomy. In all tests, p-values <0.05 were considered statistically significant.

### Patients

From the 3,261 patients initially included in IHC analyses, follow-up data was available for 2,927 patients (89.8%) with a median observation period of 84.4 months (range 1 to 219 months; see Supplemental Table 3 for baseline characteristics).

### Ethical statement

Use of tissue samples within the study was approved by the Ethics Commission of Hamburg, Germany (WF-049/09 and PV3652) and conducted in accordance with the Declaration of Helsinki. The need for informed consent was waived as all used samples originate from routine diagnostic workup. Usage of routinely archived leftover formalin fixed diagnostic tissue samples for research purposes by the attending physician is approved by local laws and does not require written consent (HmbKHG, §12,1). However, informed consent for the general use of leftover diagnostic blood and tissue samples – but not for individual studies specifically - is obtained routinely during admission to our center since 2009.

## Supplementary information

Supplementary Information.

## Data Availability

The datasets analyzed during the current study are available in the Gene Expression Omnibus (GEO) repository under https://www.ncbi.nlm.nih.gov/sites/GDSbrowser?acc=GDS2545 and https://www.ncbi.nlm.nih.gov/sites/GDSbrowser?acc=GDS4109.
