# Supplementary material for: Subcellular Compartmentalization of Survivin is Associated with Biological Aggressiveness and Prognosis in Prostate Cancer
Source: Sci Rep. 2020 Feb 24;10:3250. doi: 10.1038/s41598-020-60064-9 (PMC7039909; doi:10.1038/s41598-020-60064-9)
Supplement: Supplementary file 1 — Supplementary Information. [file 41598_2020_60064_MOESM1_ESM.pdf]

SUPPLEMENTAL INFO

Subcellular Compartmentalization of Survivin is Associated with Biological Aggressiveness and Prognosis in Prostate Cancer

Jan K. Hennigs, Sarah Minner, Pierre Tennstedt, Rolf Löser, Hartwig Huland, Hans Klose, Markus Graefen, Thorsten Schlomm, Guido Sauter Carsten Bokemeyer, and Friedemann Honecker

Supplemental Table 1:

Survivin staining intensities and frequencies in patients with radical prostatectomy by subcellular localization.

Nuclear Staining Intensity		Cytoplasmic Staining Intensity negative	weak	moderate	strong	
**negative**	n=	11	0	1	0	12
	of total	0.5%	0%	0.04%	0%	0.5%
**weak**	n=	300	190	44	3	537
	of total	13.3%	8.4%	2.0%	0.1%	23.9%
**moderate**	n=	403	280	255	90	1028
	of total	17.9%	12.4%	11.3%	4.0%	45.7%
**strong**	n=	192	62	319	100	673
	of total	8.5%	2.8%	14.2%	4.4%	29.9%
		906	532	619	193	2550
p<0.0001		40.3%	23.6%	27.5%	8.6%	100%

Pearson's χ^2 test

Supplemental Table 2:

Nuclear survivin staining intensities and frequencies in patients with radical prostatectomy

Parameter	Variable	n =	negative (%)	weak (%)	moderate (%)	strong (%)	P value*
Gleason score	≤ 3 + 3	891	0.9	28.6	45.7	24.8	<0.0001
	3 + 4	950	0.4	21.4	45.6	32.6	
	4 + 3	243	0.0	19.8	45.7	34.6	
	≥4 +4	48	0.0	12.5	52.1	35.4	
pT stage	pT2	1,329	0.8	25.5	46.1	27.6	0.0268
	pT3a	474	0.2	19.6	45.4	34.8	
	pT3b	296	0.3	23.3	44.3	32.1	
	pT4	32	0.0	34.4	50.0	15.6	
Surgical Margin Status	Negative	1,663	0.6	25.0	45.5	28.9	0.1964
	Positive	466	0.4	20.6	46.8	32.2	
Pre-operative PSA [ng/ml]	< 4	320	0.4	26.7	48.1	24.7	0.1137
	4 - 10	1,119	0.5	24.0	46.0	29.5	
	10 – 20	473	0.6	20.5	44.6	34.3	
	> 20	184	1.1	29.4	42.4	27.2	

Deviations from total are due to missing data in the subcategories.
* Pearson's χ^2 test.

Supplemental Table 3: Baseline Characteristics of 2,250 prostate cancer patients included in the IHC study*

**Parameter**	**Variable**	**n =**	**%**
Age at diagnosis (y)	<50	54	2.6
	50-60	669	31.8
	60-70	1,255	59.7
	>70	124	5.9
Pre-operative PSA [ng/ml]	< 4	320	15.2
	4 - 10	1,122	53.4
	10 – 20	474	22.6
	> 20	184	8.8
Gleason score	≤ 3 + 3	891	41.7
	3 + 4	953	44.6
	4 + 3	244	11.4
	≥4 +4	48	2.2
Pathological classification of primary tumor	pT2	1,331	62.3
	pT3a	476	22.3
	pT3b	296	13.9
	pT4	32	1.5
Pathological lymph node classification	pN0	1,090	51.2
	pN+	73	3.4
	pNx	967	45.4
Surgical margins	Negative	1,666	78.1
	Positive	467	21.9
Total:		2,250*	

*Deviations from total are due to missing data in the subcategories

Survivin IHC in Normal Adjacent Tissue

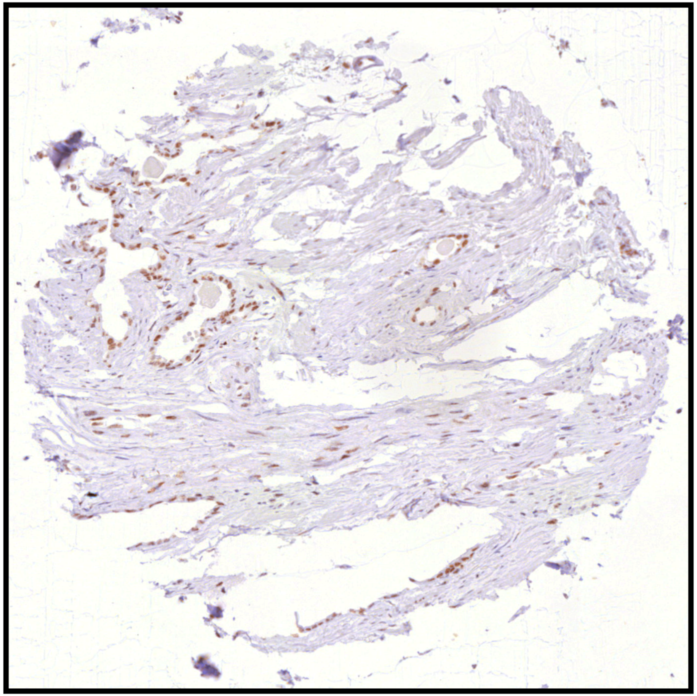

SUPPLEMENTAL FIGURE 1: Survivin immunohistochemistry in non-cancerous prostate tissue

Microphotograph of a representative TMA spot showing exclusive nuclear survivin staining in non-cancerous prostate tissue adjacent to prostate cancers.

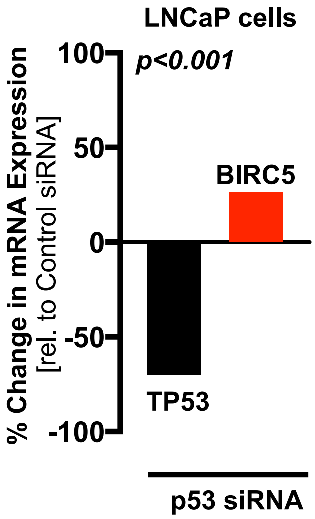

SUPPLEMENTAL FIGURE 2: Transcriptional interaction

Knockdown of p53 by siRNA (-70% p53 mRNA) in the androgen-sensitive human prostate adenocarcinoma cell line LNCaP significantly induced BIRC5 expression by 27% (GEO GSE66977 dataset, LIMMA analysis, n=6).
